# Metallurgical Tests in Endodontics: A Narrative Review

**DOI:** 10.3390/bioengineering9010030

**Published:** 2022-01-12

**Authors:** Alessio Zanza, Marco Seracchiani, Rodolfo Reda, Gabriele Miccoli, Luca Testarelli, Dario Di Nardo

**Affiliations:** Department of Oral and Maxillofacial Sciences, La Sapienza University of Rome, 00161 Rome, Italy; alessio.zanza@uniroma1.it (A.Z.); marco.seracchiani@uniroma1.it (M.S.); gabriele.miccoli@uniroma1.it (G.M.); luca.testarelli@uniroma1.it (L.T.); dario.dinardo@uniroma1.it (D.D.N.)

**Keywords:** endodontics, endodontic rotary instruments, metallurgy, nickel–titanium alloy, root canal treatment

## Abstract

Since there are no reviews of the literature on this theme, the aim of this narrative review is to summarize the metallurgical tests used in endodontics, pointing out their functional use and their pros and cons and giving readers a user-friendly guide to serve as an orientation aid in the plethora of metallurgical tests. With this purpose, a literature search for articles published between January 2001 and December 2021 was conducted, using the electronic database PubMed to collect all published articles regarding the metallurgical tests used in endodontics for the evaluation of NiTi rotary instruments. The search was conducted using the following keywords: “metallurgy”, “differential scanning calorimetry” (DSC), “X-ray diffraction” (XRD), “atomic force microscopy” (AFM), “energy-dispersive X-ray spectroscopy” (EDS), “focused ion beam analysis” (FIB) and “Auger electron spectroscopy” (AES) combined with the term “endodontics” or “NiTi rotary instruments”. Considering the inclusion and exclusion criteria, of the 248 articles found, only 81 were included in the narrative review. According to the results, more than 50% of the selected articles were published in one of the two most relevant journals in endodontics: *International Endodontic Journal* (22.2%) and *Journal of Endodontics* (29.6%). The most popular metallurgical test was DSC, with 43 related articles, followed by EDS (33 articles), AFM (22 articles) and XRD (21 articles). Few studies were conducted using other tests such as FIB (2 articles), micro-Raman spectroscopy (4 articles), metallographic analysis (7 articles) and Auger electron spectroscopy (2 articles).

## 1. Introduction

It could be stated that the modern era of endodontics began after the introduction of the nickel–titanium (NiTi) alloy as the material of choice for the manufacturing of endodontic instruments [1]. From that moment, the way of conceiving endodontics changed considerably, although its basic principles have remained the same and could be mostly summarized in three key processes: chemo-mechanically disinfecting the root canal system to achieve a reduction in bacteria as far as possible; obtaining a stable root canal filling to entomb residual bacteria and to isolate the endodontic system from the periapical tissues; obtaining a stable coronal restoration to avoid secondary infections of the root canal system [2,3]. 

The widespread use of NiTi rotary instruments, which have almost completely replaced stainless-steel (SS) manual instruments, fundamentally arose from the two most characteristic features of NiTi alloy: superelasticity and the shape memory effect. The former is defined as the ability of the alloy to store stress up to 8% without being plastically deformed, remaining in the elastic region of deformation by the creation of a stress-induced phase, called stress-induced martensite (SIM) [4,5]. The latter allows NiTi alloy to “memorize” a pre-imposed form and to return to it on heating, as a consequence of the transition from the martensitic crystallographic phase to the austenitic phase [6,7].

In the light of the above, NiTi rotary instruments have allowed the simplification of shaping procedures, increasing the predictability and the effectiveness of endodontic treatments due to the possibility of manufacturing endodontic instruments with a greater taper and using them at higher speed without compromising their flexibility and their mechanical behavior, in a way that is not possible for SS instruments [8,9,10,11].

To date, the mechanical properties of NiTi instruments have been investigated in detail and summarized in several reviews of the literature [5,7,12], most recently in the review by Zanza et al. [13]. This review emphasized the mechanical behavior of NiTi instruments during instrumentation procedures, highlighting the current knowledge regarding cyclic fatigue and torsional resistance, flexibility, centering and shaping ability and canal transportation of endodontic instruments and criticizing the most common testing devices and their limitations. As pointed out by the authors, the main drawback of the published articles was the limited analysis performed to compare NiTi systems. In most cases, in fact, the authors performed only static tests, failing to consider the dynamic behavior of NiTi instruments and drawing misleading conclusions [13]. Moreover, in the majority of the published articles, the mechanical evaluation of NiTi instruments was not accompanied by metallurgical tests, giving only a partial understanding of the topic. For this reason, Silva et al. proposed a multimethod approach for the evaluation of different instruments, in which not only the static behavior but also the dynamicity and the metallurgical features of endodontic instruments were investigated, guaranteeing more relevant results with clinical significance [14]. Moreover, the metallurgical investigation of NiTi rotary instruments is fundamental for an in-depth comprehension of their mechanical properties, and it allows clinicians, researchers and engineers to understand the chemo-physical rationale behind certain mechanical behaviors of endodontic instruments, improving knowledge on this theme. The endodontic literature is full of mechanical comparisons between different instruments [13], with authors trying to determine which is the best-performing instrument in terms of cyclic fatigue resistance, torsional resistance, shaping ability, centering ability and cutting efficiency; however, as demonstrated in the previously mentioned reviews, these results are in most cases isolated from an in-depth investigation of the metallurgical properties of the instruments, giving only a partial view of the results of mechanical testing of instruments and their performance.

Despite the importance of metallurgical tests in endodontics, to date there are no reviews of the literature on this topic, despite the fact that in recent years it has increasingly captured the attention of researchers. 

Therefore, the aim of this narrative review was to summarize the metallurgical tests used in endodontics, pointing out their functional use, their pros and cons and the future perspectives, in order to offer readers a user-friendly guide to navigating the topic of endodontic metallurgy with ease, since this field may be tricky to understand for endodontists. Moreover, this review, in addition to the review published by Zanza et al. in a previous issue of this journal, aims to complete the general overview of the current tests used in endodontics, in terms of both the mechanical and the metallurgical behavior of NiTi rotary instruments [13].

## 2. Materials and Methods

An online search was conducted in the peer-review journals listed in PubMed to retrieve in vitro and laboratory studies regarding investigations into the metallurgical characteristics of NiTi endodontic rotary instruments, using the following search query: (“metallurgic” [All Fields] OR “metallurgical” [All Fields] OR “metallurgically” [All Fields]) AND (“endodontal” [All Fields] OR “endodontic” [All Fields] OR “endodontical” [All Fields] OR “endodontically” [All Fields] OR “endodontics” [MeSH Terms] OR “endodontics” [All Fields]) AND 2001/01/01:3000/12/31 [Date—Publication].

Then, a specific search was performed for each metallurgical test evidenced in the above-mentioned search (i.e., differential scanning calorimetry (DSC), X-ray diffraction (XRD), atomic force microscopy (AFM), energy-dispersive X-ray spectroscopy (EDS or EDX), focused ion beam analysis (FIB) and Auger electron spectroscopy (AES)), respectively, using the following search queries: (“calorimetry, differential scanning” [MeSH Terms] OR (“calorimetry” [All Fields] AND “differential” [All Fields] AND “scanning” [All Fields]) OR “differential scanning calorimetry” [All Fields] OR (“differential” [All Fields] AND “scanning” [All Fields] AND “calorimetry” [All Fields])) AND ((“nitinol” [Supplementary Concept] OR “nitinol” [All Fields] OR “nickel titanium” [All Fields]) AND (“rotaries” [All Fields] OR “rotary” [All Fields]) AND (“instrument” [All Fields] OR “instrument s” [All Fields] OR “instrumentation” [MeSH Subheading] OR “instrumentation” [All Fields] OR “instruments” [All Fields] OR “instrumented” [All Fields] OR “instrumenting” [All Fields])) AND 2001/01/01:3000/12/31 [Date—Publication]; (“x ray diffraction” [MeSH Terms] OR (“x ray” [All Fields] AND “diffraction” [All Fields]) OR “x ray diffraction” [All Fields] OR “x ray diffraction” [All Fields]) AND ((“nitinol” [Supplementary Concept] OR “nitinol” [All Fields] OR “nickel titanium” [All Fields]) AND (“rotaries” [All Fields] OR “rotary” [All Fields]) AND (“instrument” [All Fields] OR “instrument s” [All Fields] OR “instrumentation” [MeSH Subheading] OR “instrumentation” [All Fields] OR “instruments” [All Fields] OR “instrumented” [All Fields] OR “instrumenting” [All Fields])) AND 2001/01/01:3000/12/31 [Date—Publication]; (“spectrometry, x ray emission” [MeSH Terms] OR (“spectrometry” [All Fields] AND “x ray” [All Fields] AND “emission” [All Fields]) OR “x-ray emission spectrometry” [All Fields] OR “energy dispersive x ray spectroscopy” [All Fields]) AND ((“nitinol” [Supplementary Concept] OR “nitinol” [All Fields] OR “nickel titanium” [All Fields]) AND (“rotaries” [All Fields] OR “rotary” [All Fields]) AND (“instrument” [All Fields] OR “instrument s” [All Fields] OR “instrumentation” [MeSH Subheading] OR “instrumentation” [All Fields] OR “instruments” [All Fields] OR “instrumented” [All Fields] OR “instrumenting” [All Fields])) AND 2001/01/01:3000/12/31 [Date—Publication]; (“accommodation, ocular” [MeSH Terms] OR (“accommodation” [All Fields] AND “ocular” [All Fields]) OR “ocular accommodation” [All Fields] OR “focusing” [All Fields] OR “focused” [All Fields] OR “focuses” [All Fields]) AND (“ions” [MeSH Terms] OR “ions” [All Fields] OR “ion” [All Fields]) AND “beam” [All Fields] AND (“endodontal” [All Fields] OR “endodontic” [All Fields] OR “endodontical” [All Fields] OR “endodontically” [All Fields] OR “endodontics” [MeSH Terms] OR “endodontics” [All Fields]) AND 2001/01/01:3000/12/31 [Date—Publication]; (“auger” [All Fields] OR “augers” [All Fields]) AND (“electron s” [All Fields] OR “electrone” [All Fields] OR “electrons” [MeSH Terms] OR “electrons” [All Fields] OR “electron” [All Fields]) AND (“spectroscopies” [All Fields] OR “spectroscopy s” [All Fields] OR “spectrum analysis” [MeSH Terms] OR (“spectrum” [All Fields] AND “analysis” [All Fields]) OR “spectrum analysis” [All Fields] OR “spectroscopy” [All Fields]) AND (“endodontal” [All Fields] OR “endodontic” [All Fields] OR “endodontical” [All Fields] OR “endodontically” [All Fields] OR “endodontics” [MeSH Terms] OR “endodontics” [All Fields]) AND 2001/01/01:3000/12/31 [Date—Publication]; (“photoelectron spectroscopy” [MeSH Terms] OR (“photoelectron” [All Fields] AND “spectroscopy” [All Fields]) OR “photoelectron spectroscopy” [All Fields] OR “x ray photoelectron spectroscopy” [All Fields]) AND (“endodontal” [All Fields] OR “endodontic” [All Fields] OR “endodontical” [All Fields] OR “endodontically” [All Fields] OR “endodontics” [MeSH Terms] OR “endodontics” [All Fields]) AND 2001/01/01:3000/12/31 [Date—Publication]; (“microscopy, atomic force” [MeSH Terms] OR (“microscopy” [All Fields] AND “atomic” [All Fields] AND “force” [All Fields]) OR “atomic force microscopy” [All Fields] OR (“atomic” [All Fields] AND “force” [All Fields] AND “microscopy” [All Fields])) AND (“endodontal” [All Fields] OR “endodontic” [All Fields] OR “endodontical” [All Fields] OR “endodontically” [All Fields] OR “endodontics” [MeSH Terms] OR “endodontics” [All Fields]) AND 2001/01/01:3000/12/31 [Date—Publication].

### 2.1. Inclusion and Exclusion Criteria

The inclusion criteria for this review were articles from peer-reviewed journals indexed in PubMed and written in English from January 2001 to December 2021 that reported at least one in vitro or laboratory test regarding the investigation of metallurgical characteristics of endodontic instruments. Thus, studies that did not meet the above inclusion criteria, such as articles not written in English, published before January 2001 or not reporting a metallurgical test of NiTi rotary endodontic instruments were excluded. Moreover, all articles in which the metallurgical tests were performed on a wire blank and not on manufactured endodontic instruments were also excluded, as well as articles on SS manual instruments.

### 2.2. Search Methodology

The titles and abstracts of all articles identified from the electronic searches in PubMed were examined by two authors (A.Z. and R.R.) in order to eliminate, in the first instance, articles that clearly failed to meet the inclusion criteria. Full-text copies of all remaining articles were further examined independently by both authors to establish whether the inclusion criteria were met. The investigators met and reviewed the remaining list of articles.

## 3. Results

The final list of articles generated after electronic searching included 248 studies. After the first screening and the discarding of duplications, 137 of these articles were obtained for full-text review. After full-text review, a total of 81 articles were included (Table 1).

**Figure 1:**
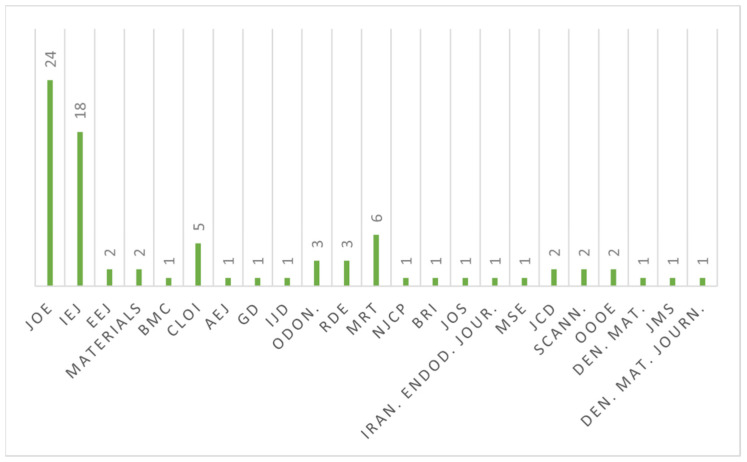
Schematic representation of the 81 articles according to publication journals. JOE (Journal of Endodontics); IEJ (International Endodontic Journal); EEJ (European Endodontic Journal), BMC (BMC Oral Health); CLOI (Clinical Oral Investigation); AEJ (Australian Endodontic Journal); GD (General Dentistry); IJD (International Journal of Dentistry); Odon. (Odontology); RDE (Restorative Dentistry and Endodontics); MRT (Microscopy Research and Technique); NJCP (Nigerian Journal of Clinical Practice); BRI (BioMed Research International); JOS (Journal of Oral Science); Iran. Endod. Jour. (Iranian Endodontic Journal); MSE (Materials Sciences and Engineering); JCD (Journal of Conservative Dentistry); Scann. (Scanning); OOOE (Oral Surgery, Oral Medicine, Oral Pathology, Oral Radiology, and Endodontology); Den. Mat. (Dental Materials); JMS (Journal of Materials Science: Materials in Medicine); Den. Mat. Journ (Dental Materials Journal).

Of those 81 articles, 45 articles were published in the two most relevant journals regarding endodontics: *International Endodontic Journal* with 19 articles (22.2%) and *Journal of Endodontics* with 26 articles (29.6%) (Figure 1).

**Figure 2:**
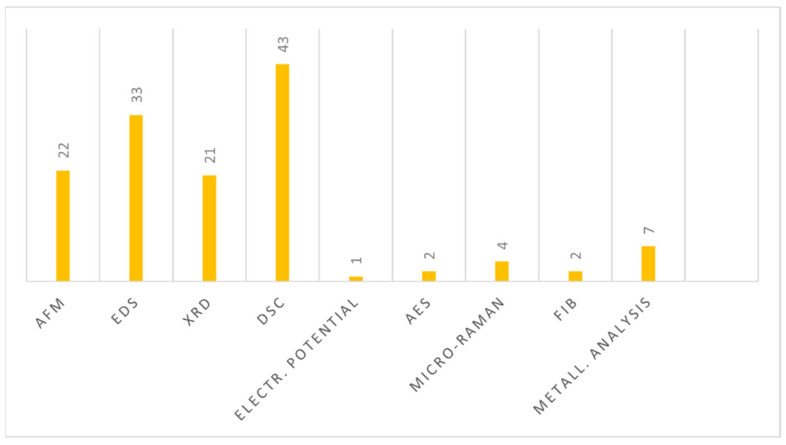
Schematic representation of the metallurgical tests used in the 81 selected articles from 2001 to 2021. XRD (X-ray diffraction), DSC (differential scanning calorimetry), EDS (energy-dispersive X-ray spectroscopy), FIB (focused ion beam analysis), AES (Auger electron spectroscopy), XPS (X-ray photoelectron spectroscopy) and AFM (atomic force microscopy); electr. potential (electrochemical potential measurement); metal. analysis (metallographic analysis).

According to the collected articles, the most popular metallurgical investigation was DSC, followed by EDS, AFM, XRD, metallurgical analysis, micro-Raman spectroscopy, FIB, AES and electrochemical potential measurements (Figure 2). 

## 4. Discussion

In the last decades, NiTi rotary instruments have become the most widespread endodontic instruments, increasing their use over SS manual instruments. This is mainly due to their undisputable advantages in comparison to SS instruments, i.e., the superelastic behavior and the shape memory effect of NiTi alloys that allow the manufacturing of endodontic instruments with a greater taper, cutting efficiency and performance. All these characteristics guarantee the simplification of instrumentation procedures, an increase in the predictability and effectiveness of endodontic treatments and a reduction in the time of endodontic treatments [5,7,12,13,95,96,97,98,99]. Regarding the increased performance of NiTi rotary instruments and their mechanical behavior, several studies have been published and summarized in many literature reviews [5,7,12,13]. Nevertheless, to date, there are no literature reviews regarding the metallurgy of NiTi rotary instruments and the metallurgical tests used in endodontics. For this reason, the aim of this narrative review was to summarize all the tests used in endodontics and to investigate the properties of NiTi alloy, giving readers and researchers an overview of this theme.

The metallurgy of endodontic instruments is fundamental for an in-depth understanding of their mechanical behavior, for the evaluation of recently introduced instruments and for comparisons between them. Unsurprisingly, of the 81 selected articles, more than 50% were published in one of the two most relevant journals in endodontics (JOE and IEJ), demonstrating the importance of metallurgical tests in addition to mechanical tests when a comparison between instruments is performed. Therefore, to the best of our knowledge, there is no equal correspondence between articles related to the comparison between different instruments and articles in which metallurgical tests were performed. In fact, performing a literature search in PubMed regarding articles on mechanical evaluation of NiTi rotary instruments using the keywords “(cyclic fatigue OR shaping ability OR torsional resistance OR flexibility OR centering ability OR cutting efficiency) AND endodontic rotary instruments” led to a total of about 670 articles being found. Comparing these results with the number of articles regarding the metallurgical investigation of NiTi rotary instruments, it is clear that in most cases the comparison between endodontic instruments was performed using only mechanical evaluation and not considering an investigation of their metallurgy. This could lead to a partial and limited view of the topic and in some cases to misleading conclusions. In our opinion, in order to improve the quality of the research, one or more metallurgical tests should always be performed when researchers intend to compare different instruments, thus performing multimethod research that investigates the whole topic. Probably, the lack of articles on the metallurgy of endodontic rotary instruments is due to two main factors: the high cost of the devices used for these types of tests and the need for the presence of specialized technicians in these investigations. Moreover, endodontic researchers may have difficulties in choosing the most appropriate test for their aims, since metallurgy is not their field of expertise. Hence, the aim of this narrative review was to summarize the metallurgical tests used in endodontics and to offer the readers a user-friendly guide to navigating the topic of endodontic metallurgy with ease, helping researchers to choose the most appropriate test for their scope.

The following is a list of all the metallurgical tests performed for the evaluation of NiTi rotary instruments from January 2001 to December 2021.

### 4.1. Differential Scanning Calorimetry

The most commonly used metallurgical test in endodontics was undoubtedly DSC. The pioneering research that introduced it for the first time in the endodontic field was conducted by Brantley et al. in 2002 [91]. DSC, as stated by the authors, was used to evaluate the structure of the NiTi alloys via accurate measurement of the difference in thermal power supplied to a test specimen and an inert control specimen heated at the same rate. More precisely, both specimens were cooled to temperatures ranging from −70 °C to −130 °C and subsequently underwent a heating/cooling cycle in the range of about −130 °C to 110 °C, with an isotherm step at 100–110 °C for one minute. Structural transformations and transition temperatures in the NiTi alloys were revealed as endothermic peaks on the heating DSC curves and as exothermic peaks on the cooling DSC curves. Moreover, DSC provides the enthalpy change during transformation and the temperature ranges of the transformation between the three crystallographic phases: austenite, R-phase and martensite [91]. One of the key phases of DSC analysis is the specimen preparation. The pans (aluminum or SS) are not able to contain the whole length of instruments; thus, they must be cut into parts and then placed in the pans. The cutting must be performed under a constant water-cooling jet and with a slow-speed diamond bur to minimize mechanical stresses that might change the crystallographic phase of the NiTi instruments [84]. 

DSC was mainly used to determine the enthalpy changes and transition temperature (martensitic transformation starting point (Ms), martensitic transformation finishing point (Mf), reverse transformation starting point (As) and reverse transformation finishing point (Af)); however, it was also used to investigate the modification of the NiTi alloy after clinical use. The results clearly showed that the enthalpy changes of as-received instruments were higher than those of instruments with simulated clinical use, showing that instruments used during instrumentation procedures are subjected to considerable stresses and are significantly changed in microstructure [18,90]. Moreover, the stored stress after instrumentation significantly modifies the transition temperatures, reducing the Af and Mf temperatures and increasing the Ms temperature [65,90].

### 4.2. Energy-Dispersive X-ray Spectroscopy 

X-ray energy-dispersive spectroscopy (EDS) is an analytical technique used for the elemental analysis or chemical characterization of a sample and was used to identify and characterize semi-quantitatively the chemical elements of the NiTi alloy and the presence of deposits on the instrument surfaces. Therefore, EDS was used to determine the near-equiatomic composition of NiTi instruments and to detect any other materials present as inclusions in the alloy or as adherent deposits on the surface [76,93]. 

In the last decades, EDS was mainly used to determine the importance of differences in terms of percentages of Ni and Ti in the alloy used for the manufacture of NiTi rotary instruments; however, its relevance is still dubious. Alapati et al. reported that differences in the wire blanks in terms of percentages of Ni and Ti, in addition to the machining procedures, can alter the metallurgical characteristics of NiTi instruments and can influence their mechanical performance [75]. However, the potential role of different weight percentages of nickel still remains uncertain, since there are no studies that evaluate the influence of this factor on the mechanical properties of the NiTi instrument while maintaining unchanged all other factors such as instrument design and heat treatment. Thus, it is difficult to determine whether there is a crucial effect of a single factor (Ni and Ti percentage) or as is more likely, a combination of different correlated factors, when determining the final mechanical properties. Furthermore, Zinelis et al. reported that the thermomechanical history, as well as the instrument design, has a much more crucial effect on the final mechanical behavior in comparison to the compositional deviations of NiTi instruments [73]. 

EDS analysis has also been used to investigate the composition of the as-received NiTi instrument surface and its variation after clinical use, cycles of sterilization or immersion in endodontic solutions. Several studies demonstrated that the manufacturing procedures of NiTi instruments, such as machining operations and heating of the alloy, lead to the formation of irregularities and cause the presence of adherent material on the instrument’s surface, to which dentine adheres after root canal shaping [4,76,93]. For this reason, particular attention during cleaning procedures before sterilization or the single use of endodontic instruments is necessary to reduce the possibility of cross contamination, since the adherence of dentine to the deposits may prevent appropriate sterilization of NiTi instruments and enhance the risk of cross infection between patients [59,93].

Moreover, Spagnuolo et al. evaluated the effect of autoclaving cycles on the chemical composition of NiTi alloy, stating that the percentage of nickel and titanium was affected by these procedures, with a decrease in nickel and titanium for ProTaper Universal and a decrease in nitrogen and titanium with an increase in nickel for AlphaKyte [67]. As mentioned before, the immersion of NiTi instruments in endodontic solutions could also alter their surface composition, leaving sodium and chlorine elements on the surface, with a consequent decrease in the weight percentage of nickel and titanium and a corresponding increase in the weight percentage of sodium and chlorine elements [58]. However, to date, there are no data on the influence of these surface changes on the mechanical behavior of endodontic instruments. Thus, further studies are required.

Hence, DSC, EDS and XRD should be considered as important types of analysis in completely understanding the different mechanical behaviors of NiTi rotary instruments.

### 4.3. Atomic Force Microscopy

Atomic force microscopy was introduced for the first time as a tool for the evaluation of NiTi instrument surfaces by Valois et al. [86]. Its advantages are fundamentally its ease of use, low cost and molecular-level resolution of structural detail, providing three-dimensional images with high spatial resolution, as demonstrated by several studies using AFM [100,101]. Qualitative and quantitative information on the NiTi instrument’s topography are provided via the detection of several forces between the probing tip used in this analysis and the sample [86]. For this reason, AFM could be considered as part of the scanning probe microscopy family, with the ability to reconstruct the surfaces of NiTi instruments three-dimensionally (as sets of x, y and z values). These sets are then analyzed with dedicated digital software to give all the data pertaining to the examined surface in quantitative form, using vertical topographic parameters [79,100,102]. The most widely used evaluation parameters for describing the topographic characteristics of a surface are the arithmetic mean roughness (AMR), maximum height (MH) and root mean square (RMS) [79,82,86].

AFM was used to evaluate the surfaces irregularities of as-received endodontic instruments and their changes after clinical use. Valois et al. stated that all tested instruments showed surface irregularities arising from the manufacturing procedure [86], and this has been confirmed by other articles published in subsequent years [45,63]. Moreover, it was stated that root canal shaping and the consequent multiple autoclave cycles can cause the deformation of NiTi instrument surfaces, increasing their irregularities and thus their roughness [42,79,82].

Another potential area of application of AFM was the determination of surface irregularities of NiTi instruments caused by their immersion in irrigating solutions such as sodium hypochlorite (NaOCl) and ethylenediaminetetraacetic acid (EDTA). Ametrano et al., Saglam et al., Fayyad et al. and Uslu et al. reported that the immersion of NiTi instruments in NaOCl caused a slight but significant degradation of the surface, increasing their roughness. Although with different intensities, even EDTA and chlorhexidine affect the instrument’s surface regularity [16,41,64,68,71].

### 4.4. X-ray Diffraction

X-ray diffraction (XRD) is one of the most valuable tools for the determination of the crystallographic structure of materials, in particular for NiTi alloy. XRD and DSC are able to provide a complete description of the NiTi alloy, both in terms of crystallographic organization (austenite, martensite and R-phase) and transition temperatures between each phase. Khun et al. explained in detail the operation of XRD analysis as follows:

*“Planes (hkl) of atoms constructively interfere with X-rays and diffraction occurs: Bragg’s law, λ = 2d sinθ, allows the calculation of interplanar spacings or d-spacings from the angular location of XRD peaks, θ (degree). Comparison of XRD data to known standards is used to identify phases. Miller indices, hkl integers, are assigned to XRD peaks. Miller indices describe the orientation of planes of atoms to the unit cell of a material’s crystal structure”*

[94]

Thus, XRD is mainly used to evaluate the crystallographic phase organization of the NiTi alloy of endodontic instruments and to assess the influence of heat treatments on the constitution of the phases in the instruments. However, the main disadvantage of this test is the impossibility of establishing the amount of each phase in the samples with sufficient precision. In other words, is not possible to know the exact percentage of austenite, martensite and R-phase in NiTi rotary instruments; only the most represented crystallographic organization of NiTi [33].

### 4.5. Metallographic Analysis

A metallographic analysis is usually performed using scanning electron microscopy to identify the grains of austenite and martensite in the NiTi alloy. Thus, this kind of test is fundamentally used to confirm and complete the results of XRD and EDS and to detect any inclusions in the alloy. In order to disclose the microstructure of the NiTi matrix, samples must be embedded in epoxy resin, ground, polished and then etched with 60% nitric acid, 10% fluorhydric acid and 30% acetic acid at room temperature for 5 s [103]. However, Generali et al. proposed a different method for the preparation of the specimen for metallographic analysis in order to reduce the artefacts that might have arisen from metallographic etching with hydrofluoric-acid-based compounds, that consisted of vibro-polishing and plasma-cleaning of the resin-mounted specimen instead of the etching procedure [28].

### 4.6. Micro-Raman Spectroscopy, Focused Ion Beam Analysis and Auger Electron Spectroscopy

Micro-Raman spectroscopy and focused ion beam analysis were methods used to investigate the surface of NiTi instruments to assess the presence of surface layers or coatings. In the endodontic literature, there were only four studies that used micro-Raman spectroscopy and two studies that used FIB analysis in their methodology, and all of these were published by the same research group [22,26,28,44]. 

Auger electron spectroscopy is a through-thickness method that is based on ion bombardment. It is able to evaluate the chemical composition of the alloy in the surface layers, up to a depth of a few micrometers, in contrast to FIB/SEM and micro-Raman analyses [26].

## 5. Conclusions

Metallurgical tests are fundamental for thoroughly understanding the mechanical behavior of NiTi rotary instruments, particularly when the aim of the study is to compare the performance of different instruments. Moreover, they allow clinicians, researchers and engineers to understand the chemo-physical rationale behind certain mechanical behaviors of endodontic instruments, giving further information for developing knowledge in this field.

## Figures and Tables

**Table 1:** Summary of all articles that met the inclusion criteria, in chronological order from more recent to older. XRD (X-ray diffraction), DSC (differential scanning calorimetry), EDS (energy-dispersive X-ray spectroscopy), FIB (focused ion beam analysis), AES (Auger electron spectroscopy), XPS (X-ray photoelectron spectroscopy) and AFM (atomic force microscopy).

First Author and Publication Year	Journal	Metallurgical Tests	Selected Instruments
Martins (2021) [15]	International Endodontic Journal	DSC and EDS	ProTaper Next and X-File
Jose (2021) [16]	European Endodontic Journal	AFM and EDS	XP-endo Shaper and TruNatomy
Jo (2021) [17]	Materials	DSC	OneShape, OneCurve, WaveOne Gold and HyFlex EDM
Van Pham (2021) [18]	BMC Oral Health	DSC	Reciproc, HyFlex CM, and Neoniti A1
Martins (2021) [19]	Clinical Oral Investigations	EDS and DSC	ProTaper Universal, U-File, ProTaper Gold, Premium Taper Gold, Go-Taper Flex,
Kalyoncuoğlu (2021) [20]	Australian Endodontic Journal	XRD and DSC	Reciproc and Reciproc Blue
Martins (2021) [21]	International Endodontic Journal	DSC, EDS	Reciproc, Reciproc Blue, One Files, One Files Blue, Reverso Silver, and WaveOne Gold
Azizi (2021) [22]	European Endodontic Journal	EDS, micro-Raman spectroscopy, FIB, XRD, metallographic analysis and DSC (Buono)	OneShape and OneCurve
Keskin (2021) [23]	Clinical Oral Investigations	DSC	Rotate, Reciproc Blue, Reciproc, and Mtwo
Martins (2020) [24]	Journal of Endodontics	EDS and DSC	ProTaper Universal, EdgeTaper, U-File, Go-Taper Universal, Super Files, Multitaper and Pluri Taper.
Martins (2020) [25]	Journal of Endodontics	EDS and DSC	ProTaper Universal, ProTaper Gold, U-File, Super Files and Super Files Blue
Generali (2020) [26]	Materials	EDS, FIB, micro-Raman spectroscopy, XRD, DSC and AES with depth profiling	Procodile and Reziflow
Silva (2020) [14]	Journal of Endodontics	DSC and EDS	NeoNiti, HyFlex EDM, ProTaper Gold and ProTaper Universa
Weyh (2020) [27]	General Dentistry	EDS	ProTaper Gold, EdgeTaper Platinum, ProTaper Universal, EdgeTaper, Vortex Blue and EdgeSequel Sapphire.
Generali (2020) [28]	International Endodontic Journal	EDS, FIB, micro-Raman spectroscopy, metallographic analysis, XRD, DSC and depth-sensing indentation test	Reciproc and Reciproc Blue
Arias (2020) [29]	Clinical Oral Investigations	DSC	HyFlex EDM and TRUShape
Khabadze (2020) [30]	International Journal of Dentistry	EDS and XRD	ProTaper Universal
Almeida (2019) [31]	International Endodontic Journal	DSC	Reciproc and Reciproc Blue
Staffoli (2019) [32]	Odontology	DSC	One Curve and One Shape New Generation, One Shape
Garcia (2019) [33]	Journal of Endodontics	XRD and DSC	WaveOne and WaveOne Gold
Generali (2019) [34]	Odontology	EDS, DSC, metallographic analysis, XRD and depth-sensing indentation test	Endo-Eze™ Genius and Reciproc
Arias (2019) [35]	Clinical Oral Investigations	DSC	EdgeSequel Sapphire and Vortex Blue
Khalil (2019) [36]	Restorative Dentistry and Endodontics	AFM and EDS	EdgeEvolve and ProTaper Gold
Üreyen Kaya (2019) [37]	Microscopy Research and Technique	EDS and AFM	ProTaper Retreatment and WaveOne Gold
Arias (2018) [38]	Journal of Endodontics	DSC	Hyflex EDM and TRUShape
Pedullà (2018) [39]	Restorative Dentistry and Endodontics	DSC	M3 Rotary and M3 Pro Gold
Pereira (2018) [40]	International Endodontic Journal	EDS and electrochemical potential measurements	ProTaper Universal, ProTaper Next, Typhoon, Hyflex EDM and Vortex Blue
Uslu (2018) [41]	Microscopy Research and Technique	AFM	HyFlex CM and HyFlex EDM
Özyürek (2018) [42]	Restorative Dentistry and Endodontics	AFM	WaveOne and WaveOne Gold
Yılmaz (2018) [43]	Clinical Oral Investigations	AFM	HyFlex EDM and HyFlex CM
Iacono (2017) [44]	International Endodontic Journal	XRD, DSC, EDS, micro-Raman spectroscopy and depth-sensing indentation test	HyFlex EDM and HyFlex CM
Inan (2017) [45]	Nigerian Journal of Clinical Practice	AFM	Twisted Files and Mtwo
Cai (2017) [46]	International Endodontic Journal	AFM	HyFlex CM and M3 Rotary
Shim (2017) [47]	BioMed Research International	DSC	ProFile, K3, One Shape, ProTaper Next, Reciproc, WaveOne, HyFlex CM and Twisted File
Kalyoncuoğlu (2016) [48]	Journal of Oral Science	EDS	Reciproc and ProTaper Retreatment
Aminsobhani (2016) [49]	Iranian Endodontic Journal	EDS, DSC and XRD	Neoniti, iRaCe, Mtwo, Twisted File and ProTaper Next
de Vasconcelos (2016) [50]	Journal of Endodontics	DSC	ProTaper Universal, HyFlex CM, TRUShape and Vortex Blue
Aun (2016) [51]	Materials Sciences and Engineering	DSC and XRD	iRaCe
Pirani (2016) [52]	International Endodontic Journal	EDS and metallographic analysis	Hyflex EDM
Shen (2015) [53]	Journal of Endodontics	DSC	ProFile Vortex and Vortex Blue
Hieawy (2015) [54]	Journal of Endodontics	DSC	ProTaper Universal and ProTaper Gold
Nair (2015) [55]	Journal of Conservative Dentistry	AFM	Mtwo and ProTaper Universal
Can Sağlam (2015) [56]	Microscopy Research and Technique	AFM	ProTaper Retreatment, R-endo and Mtwo retreatment
Braga (2014) [57]	Journal of Endodontics	EDS and DSC	EndoWave, ProFile Vortex, HyFlex CM, Typhoon and ProTaper Universal
Prasad (2014) [58]	Journal of Conservative Dentistry	AFM and EDS	ProTaper Universal and iRaCe
Pirani (2014) [59]	Scanning	EDS and metallographic analysis	WaveOne and Reciproc
Türker (2014) [60]	Scanning	AFM	OneShape and WaveOne
Pirani (2014) [61]	Odontology	EDS and metallographic analysis	WaveOne primary and ProTaper Universal
Nakagawa (2014) [62]	International Endodontic Journal	EDS, DSC and XRD	PathFile, Race ISO 10 and Scout RaCe
Fatma (2014) [63]	Microscopy Research and Technique	AFM	ProTaper Universal, Reciproc and WaveOne
Fayyad (2014) [64]	International Endodontic Journal	AFM	ProFile GT, Twisted File, RaCe and HeroShaper
Shen (2013) [65]	International Endodontic Journal	DSC and XRD	HyFlex CM
Shen (2012) [66]	Journal of Endodontics	DSC and XRD	ProFile Vortex
Spagnuolo (2012) [67]	International Endodontic Journal	EDS and AFM	ProTaper Universal and AlphaKite
Sağlam (2012) [68]	Microscopy Research and Technique	AFM	ProTaper Universal
Yamazaki-Arasaki (2012) [69]	Microscopy Research and Technique	AFM	K3, ProTaper Universal, Twisted Files and BioRace
Shen (2011) [70]	Journal of Endodontics	DSC, XRD, EDS and metallographic analysis	EndoSequence, ProFile, ProFile Vortex, Twisted Files, Typhoon and Typhoon™ CM.
Ametrano (2011) [71]	International Endodontic Journal	AFM	ProTaper Universal
Viana (2010) [72]	Oral Surgery, Oral Medicine, Oral Pathology, Oral Radiology, and Endodontology	EDS, XRD and DSC	ProTaper Universal, K3, and EndoSequence.
Zinelis (2010) [73]	International Endodontic Journal	EDS and XRD	EndoSequence, FlexMaster, Hero 642, K3, Liberator, NRT, ProFile and ProTaper Universal
Condorelli (2010) [74]	International Endodontic Journal	XPS, EDS and XRD	RaCe
Alapati (2009) [75]	Dental Materials	XRD and DSC	ProFile GT, ProTaper Universal, K3 and Quantec
Shen (2009) [76]	Journal of Endodontics	EDS	ProFile Series 29, ProFile and ProTaper Universal
Bonaccorso (2008) [77]	Journal of Endodontics	EDS and AES	RaCe
Alves-Claro (2008) [78]	Journal of Materials Science: Materials in Medicine	XPS	Nitiflex
Valois (2008) [79]	Journal of Endodontics	AFM	Greater Taper and ProFile
Topuz (2008) [80]	Oral Surgery, Oral Medicine, Oral Pathology, Oral Radiology, and Endodontology	AFM	RaCe
Li (2007) [81]	Dental Materials Journal	XPS and DSC	ProTaper Universal
Inan (2007) [82]	Journal of Endodontics	AFM	ProTaper Universal
Hayashi (2007) [83]	International Endodontic Journal	DSC	Prototype Ni–Ti rotary instruments
Alexandrou (2006) [84]	Journal of Endodontics	DSC	ProFile and FlexMaster
Alexandrou (2006) [85]	International Endodontic Journal	DSC	NRT
Valois (2005) [86]	Journal of Endodontics	AFM	Greater Taper and Quantec
Miyai (2005) [87]	International Endodontic Journal	DSC	EndoWave, Hero 642, K3, ProFile and ProTaper Universal
Tripi (2003) [88]	Journal of Endodontics	XPS and XRD	ProFile GT
Tripi (2002) [89]	Journal of Endodontics	XPS and XRD	ProFile GT
Brantley (2002) [90]	Journal of Endodontics	DSC	ProFile and Lightspeed
Brantley (2002) [91]	Journal of Endodontics	DSC	ProFile and Lightspeed
Kuhn (2002) [92]	Journal of Endodontics	DSC	ProFile and Hero
Martins (2002) [93]	Journal of Endodontics	EDS	ProFile
Kuhn (2001) [94]	Journal of Endodontics	XRD	ProFile and Hero

## Data Availability

No new data were created or analyzed in this study. Data sharing is not applicable to this article.
